# Efficiency of Hair Detection in Hair-to-Hair Matched Trichoscopy

**DOI:** 10.1159/000524345

**Published:** 2022-05-12

**Authors:** Laita Bokhari, Phoebe Cottle, Ramon Grimalt, Michal Kasprzak, Justyna Sicińska, Rodney Sinclair, Antonella Tosti

**Affiliations:** ^a^Sinclair Dermatology, Melbourne, Victoria, Australia; ^b^Faculty of Medicine and Health Sciences, Universitat Internacional de Catalunya, UIC-Barcelona, Barcelona, Spain; ^c^TrichoLAB, Warsaw, Poland; ^d^Department of Dermatology, CSK MSWiA/CMKP Warsaw, Warsaw, Poland; ^e^Dermiq Medical Center, Warsaw, Poland; ^f^Epworth Dermatology, Melbourne, Victoria, Australia; ^g^Department of Medicine, University of Melbourne, Melbourne, Victoria, Australia; ^h^Department of Dermatology, University of Miami, Miller School of Medicine, Miami, Florida, USA

**Keywords:** Hair loss, Trichoscopy, Hair count, Hair-to-hair matching, Alopecia

## Abstract

**Introduction:**

Precise evaluation of changes in hair count is crucial for monitoring progression of hair loss and the effects of treatment. The focus of this study is the comparison of the various examination and assessment techniques in terms of the precision of hair count change observed in trichoscopy images.

**Methods:**

Controlled hair extraction of the same scalp spot was used to simulate hair loss, and the different examination techniques were performed to detect this change. The investigators who performed the counting were blinded.

**Results:**

For trichoscopy images, the average error in determining the terminal hair count change (relative to total hair count) was 9 ± 1% for automatic assessment with manual correction and 0.4 ± 0.2% for hair-to-hair matched images. For phototrichogram, the automatic measurement results were found to deviate from truth on average by 12 ± 2%. The manually corrected hair count results were much closer to the truth with average deviation at the level of 7 ± 1%. The hair-to-hair matched results corresponded to approximately 0.6 ± 0.3% average discrepancy.

**Conclusion:**

Combination of manually corrected image processing, follicular mapping, and hair-to-hair matching appears to be the most precise way of evaluating the change in hair count over time. These novel techniques should be considered valuable, especially in research and clinical trials.

## Introduction

Precise evaluation of changes in hair count are crucial for monitoring progression of hair loss and the effects of treatment [1, 2, 3, 4]. Automated software tools, manual hair counting (also called manually corrected count), and hair-to-hair (H2H) matching can all be used to statistically process microscopic hair images [5, 6]. Although some of these methods are known to measure hair count only approximately, they are believed to correctly indicate its change in before and after comparisons [7, 8, 9].

The focus of this study is the comparison of the different examination techniques in terms of the precision of the before and after hair count change. Only terminal hair shafts (thickness >40 μm) were taken into account as they form the majority of hair coverage and volume studied in most clinical and research projects.

## Materials and Methods

Healthy volunteers with contrasting hair color were selected: five from among the staff of Grimalt Dermatology, Barcelona, Spain, and four from among the staff of Sinclair Dermatology, Melbourne, Australia. The group comprised 5 females and 4 males aged 17–45 years. All subjects gave their oral consent to participate in the study in the presence of at least one witness. The subjects washed their hair the same day to remove hair that could accidentally fall out during the examinations. The different examination techniques were applied one after another on exactly the same test spot on patient's scalp. To determine the sensitivity of the techniques to hair count change, the following experiment was performed. Controlled hair extraction between subsequent examinations of the same spot was used to simulate hair loss, and the different examination techniques were performed to detect that change. In the cases of examination techniques involving human experts, a blinding procedure was used. The details of controlled hair extraction were known only to the examination team and were not disclosed to the laboratory staff responsible for image analysis, manual hair counting, or H2H matching. The clipped hair phototrichogram (PTG) and the unclipped hair trichoscopy examinations under study have been performed using the following setup:

- *Automatic PTG*: hair clipped to ca 1 mm, Dermoscan Dermogenius^TM^ videodermoscope, water immersion, TrichoScan^TM^ Version 3.7.27.124, circular measurement area of 0.59 cm^2^.
- *Manually corrected PTG*: hair clipped to ca 1 mm, FotoFinder® medicam^TM^ 1000, ×20 magnification, polarized light, TrichoLAB processing of images, rectangular measurement area of 0.78 cm^2^ area.
- *H2H matched PTG*: 3 images of the same test spot with hair clipped to ca 1 mm and re-combing between images to rearrange hair; FotoFinder medicam^TM^ 1000, ×20 magnification, polarized light, TrichoLAB F-Mapping®, and H2H Matching®;
- *Manually corrected trichoscopy*: unclipped hair, two side-by-side images registered with FotoFinder medicam^TM^ 1000, ×40 magnification, polarized light, total measurement area of 0.58 cm^2^ (examinations 1–3), FotoFinder leviacam^TM^, polarized light, measurement area of 0.98 cm^2^ (examinations 4–8), TrichoLAB processing of images;
- *H2H matched trichoscopy*: 3 pairs of unclipped hair trichoscopy images of the same spots with hair re-combed and re-parted between images, recorded with FotoFinder medicam^TM^ 1000, ×40 magnification (examinations 1–3) or FotoFinder leviacam^TM^, measurement area of 0.98 cm^2^ (examinations 4–8), TrichoLAB F-Mapping®, and H2H Matching®.

### Test Procedure

#### Trichoscopy

**Fig. 1:**
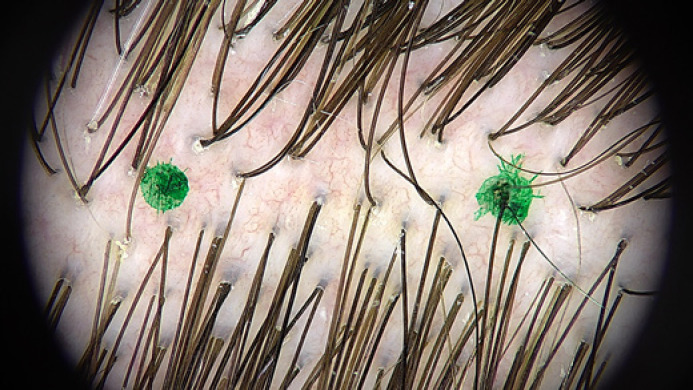
Examination spot marked with two dots 1 cm apart in subject no. 1.

1. A single examination spot was selected and marked with two felt-tip pen dots ca 1 cm apart, as illustrated in Figure 1. The same test spot was used throughout the whole examination procedure.

2. First set of trichoscopy examinations − standard trichoscopy examination and H2H matching procedures − were performed (3 sequential images of the same spot with complete rearrangement and re-parting of the hair between taking images).

3. First controlled hair removal using high magnification glasses and metal tweezers, the investigator carefully removed a few hair shafts from the test spot and made photographic documentation of the removed hair shafts and their location in relation to the two spot marking.

4. Second set of trichoscopy examinations − complete standard trichoscopy and H2H matched trichoscopy − were repeated in the same way as before the hair removal.

#### Phototrichogram

1. Hair clipping − hair from the area approximately 1.5 cm^2^ around the test spot marks was clipped to ca 1 mm and the hair remnants carefully removed.

2. First set of PTG examinations − the first image of the test spot was recorded with DermoGenius for the DermoScan analysis; subsequent images were recorded with FotoFinder medicam both for manual processing and the H2H matching PTG procedure (3 images of the same spot taken after combing of the stubble in different directions to rearrange it) [10].

3. Second controlled hair removal − the investigator removed further hair shafts from the test spot and made photographic documentation of the removed hair shafts and their location in relation to the test marks.

4. Second set of PTG examinations − the complete set of PTG images for the procedures under study were repeated in exactly the same way as before the controlled hair removal.

The PTG and the trichoscopy images obtained before and after controlled hair removal were submitted to TrichoLAB for processing. The dates/times of randomly selected examination files were modified so the lab staff could not know which was the initial one and whether to expect hair loss or hair gain. The details of controlled hair removal for each patient were known only to the examiners at Grimalt Dermatology and Sinclair Dermatology. They were disclosed only for final evaluation of examination results.

The examinations were performed at Grimalt Dermatology, Barcelona, Spain between February 20 and 26, 2019 and at Sinclair Dermatology, Melbourne, Australia between December 2, 2020 and January 20, 2021. As only 5 out of the 9 volunteer subjects agreed for hair clipping, only these subjects took part in the PTG efficiency measurement. For trichoscopy efficiency measurement, the data of 1 subject was excluded due to poor image quality.

## Results

### Trichoscopy Measurements

**Fig. 2:**
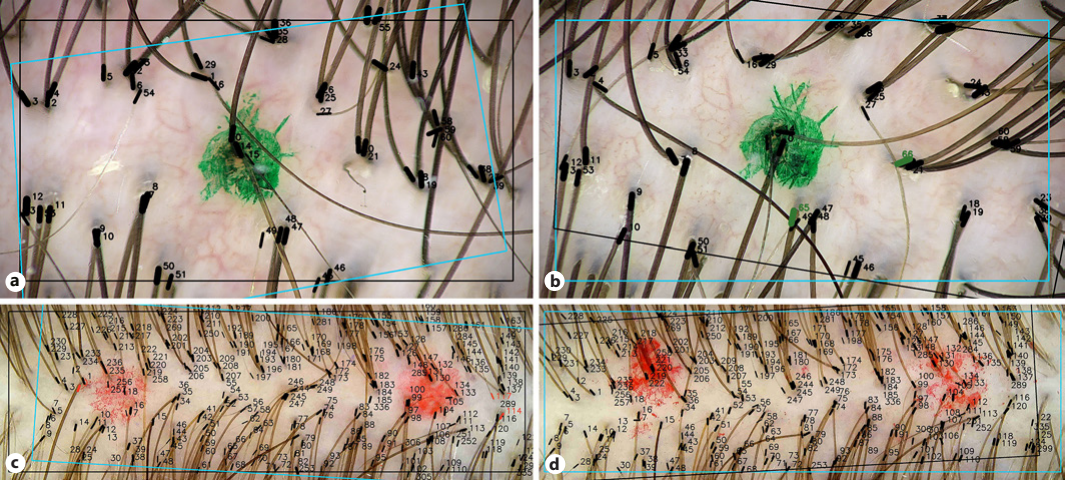
**a**H2H matched baseline image of right dot mark of subject no. 1 (FotoFinder medicam ×40). **b**H2H matched follow-up image of right dot mark of subject no. 1 (FotoFinder medicam ×40). **c**H2H matched baseline image subject no. 6 (FotoFinder leviacam). **d**H2H matched baseline image subject no. 6 (FotoFinder leviacam).

Table 1 presents the results of trichoscopy examinations: the manually corrected hair counts (columns *1*&*2*) and the H2H matching results (columns *3*&*4*) in baseline and follow-up examinations. The change in hair count derived with these two techniques is presented in columns *5* and *6*, respectively. Figure 2a, b present the H2H matched trichoscopy images of the right dot mark of subject no. 1. The two green-labelled hair shafts in Figure 2b have been detected as new hair shafts that were not present in the earlier image of Figure 2a. The images from H2H matching analysis around the left spot mark also indicated gain of two terminal hair shafts, making a total of four new hair shafts as indicated in column *6* of Table 1, accordingly. Figure 2c, d present similar images of subject no. 6 registered with FotoFinder leviacam.

### The Trichoscopy Measurement Results versus the Truth

**Fig. 3:**
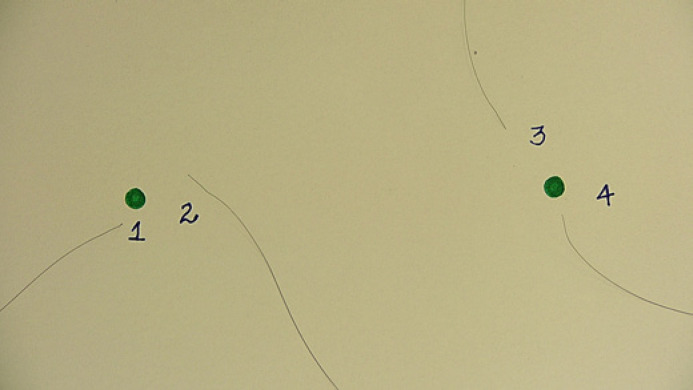
Controlled nonclipped hair extraction documentation for patient no. 1.

Once the trichoscopy examination processing was complete, the unblinding procedure was performed and column *7* was added to Table 1. It presents the number of terminal hair shafts removed in the controlled hair extraction procedure with a plus or minus sign depending on whether the baseline and follow-up examinations were swapped or not. Figure 3 shows documentation of four hair shafts extracted from subject no. 1 between the two trichoscopy examinations. As the dates of the first and the second examinations were swapped, results are presented in column *7* as a gain of four new hair shafts.

Columns *8* and *9* present the extent to which the results obtained with the two trichoscopy techniques deviate from the true change in terminal hair count. The manually corrected hair count change was found to deviate from the truth on average by 9 ± 1% (relative to the total number of measured hair). The H2H matched results were wrong in 6 cases out of 1,382 measured hair shafts, corresponding to 0.4 ± 0.2%.

### The PTG Measurements

**Fig. 4:**
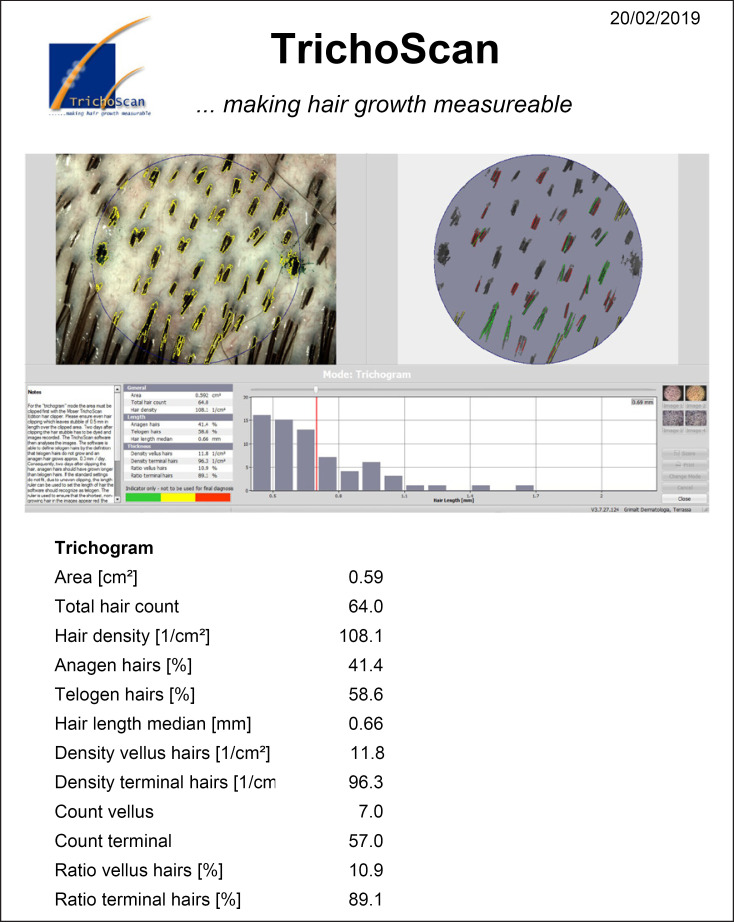
TrichoScan^TM^ report for the first PTG examination of subject no. 1.

**Fig. 5:**
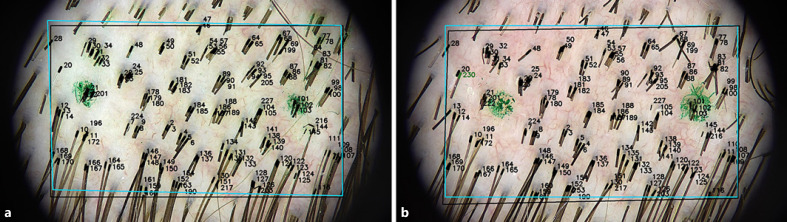
**a**H2H matched baseline PTG image for subject no. 1. **b**H2H matched follow-up PTG image for subject no. 1.

Table 2 presents the results of PTG examinations: automatic (columns *1*&*2*) and manually corrected hair counts (columns *3*&*4*) in before and after examinations as well as the H2H Matching results (columns *5*&*6*). The change in hair count derived with these three techniques is presented in columns *7, 8*, and *9*, respectively. Figure 4 presents an example of a TrichoScan^TM^ report for subject no. 1; because the standard report presents only the density of terminal hair, it was multiplied by the measurement area for the comparison. Figure 5a, b present the H2H matched PTG images for subject no. 1. Only one new hair was detected in Figure 5b (labelled green) that was not present in Figure 5a, as noted in column *9* of Table 2 accordingly.

### The PTG Measurement Results versus the Truth

**Fig. 6:**
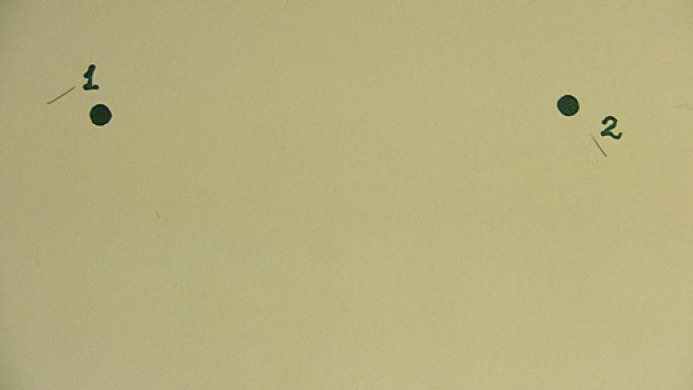
Controlled clipped hair extraction documentation for patient no. 1.

After completing the PTG examination processing, the unblinding procedure was performed and column *10* was added to Table 2. It presents the number of terminal hair shafts removed in the controlled hair extraction procedure between the two PTG examinations with a plus or minus sign depending on whether the two examinations were swapped. As presented in Figure 6, only two clipped hair shafts were extracted from subject no. 1, and the two PTG examinations were swapped. The true change presented in column *10* for patient 1 is therefore +2 hair shafts.

Columns *11, 12*, and *13* present to what extent the results obtained with the three PTG techniques deviate from the true change in terminal hair count. The automatic PTG measurement results were found to deviate from truth on average by 12 ± 2%. The manually corrected hair count results were much closer to the truth as the average deviation for the given subject sample was on the level of 7 ± 1%. The H2H matched results were wrong in 4 cases out of 724 measured hair shafts, corresponding to 0.6 ± 0.3% discrepancy.

### The Precision of the Results

The resulting detection inefficiencies are quoted with the estimation of statistical precision (after ±) derived, assuming that the number of hair shaft misdetections follows Poisson distribution. These estimates were precise enough for the purpose of this study, so it was concluded that the number of subjects was also sufficient. Analysis of systematic uncertainties generally indicated that the denser the hair patient has, the higher the multiplicities of follicular units, the more difficult it is to detect them and the larger the inefficiencies are. To address this issue, young and healthy individuals with dense hair were recruited for the study.

## Conclusion

The manually corrected PTG results are approximately twice as accurate as the automatic assessment of terminal hair count difference between the baseline and the follow-up examinations. Although more precise and reliable, manually corrected PTG suffers from similar limitations: hard to detect hair shafts sticking closely together in tight follicular units and slightly different field of measurement in the before and after examinations. The effect that was observed, but could not be studied further, is the dependency of the automatic PTG result on the clipping length; in our study, the subsequent examinations were performed with exactly the same clipping.

The H2H matched analysis of both the PTG and the trichoscopy examinations is over one order of magnitude more accurate in terms of before and after comparison. The reasons for this fact are as follows:

- The F-Mapping procedure allows recovering information about hair that could not be detected in the primary image (e.g., because their view was obstructed by other hair) from the subsequent two images of the same spot with a different hair arrangement. The F-Mapping procedure brings the effective hair detection efficiency from 90 to 95% to over 99.5%.
- Before and after matching procedure ensures that precisely the same scalp area is used for the comparison, compensating for different camera positioning as well as any skin stretching/distortions.

Automatic analysis using TrichoScan^TM^ software provides quick but precision-limited results − the measured hair count change was found to deviate from truth on average by ± 12% and the deviation could increase if the clipping in the before and after examinations was not identical. The manually corrected PTG and trichoscopy were determined to have fewer errors per 100 measured hair shafts (7% and 9% for PTG and trichoscopy, respectively) and are therefore advocated as the technique of choice when high precision is demanded, e.g., in clinical studies. The H2H matching further improves precision of the before and after comparison, reducing the number of errors to 0.4% and 0.6% for trichoscopy and PTG, respectively.

Combination of manually corrected image processing, follicular mapping, and H2H matching appears to be the most precise way of evaluating the change of hair count over time in clinical trials and hair research. Currently, as this combined procedure is time-consuming, its everyday use is rather limited. Hopefully, future developments should make this technique more available for office use.

## Statement of Ethics

All the subjects recruited from among medical staff gave their written consent to participate in this study. Ethical approval was not obtained for this study as it was not needed as per national guidelines.

## Conflict of Interest Statement

Laita Bokhari, Phoebe Cottle, and Ramon Grimalt had no conflict of interest. Michał Kasprzak is a founder and CEO of TrichoLAB. Justyna Sicińska is a consultant for TrichoLAB and FotoFinder and a family member of TrichoLAB founders.

Rodney Sinclair − Director and Founder Samson Medical Pty Ltd. Pharmaceutical advisory board Eli Lilly and Company, Pfizer Inc., Leo Pharmaceutical. Speaker bureau Abbvie, Novartis. Principal investigator in clinical trials for AbbVie, Aerotech, Akesobio, Amgen, Arcutis, Arena, Ascend AstraZeneca, Bayer AG, Biotherapeutics Boehringer Ingelheim, Bristol Myer Squibb, Celgene, Coherus BioSciences, Connect, Demira, and Eli Lilly and Company.

Galderma, Glaxo Smith Kline, F. Hoffman–La Roche, Janssen, MedImmune, Merck and Co. Merck Sharpe & Dohme, Novartis, Oncobiologics, Pfizer, Principia, Regeneron, Roche. Reistone Biopharma, Samson Clinical, Sanofi-Genzyme, Sun Pharma UCB, and Valeant and is serving as the current President of the Australasian Hair and Wool Research Society. Antonella Tosti is a consultant for DS Laboratories, Monat Global, Almirall, Tirthy Madison, Eli Lilly, Bristol Myers Squibb, P&G, Pfizer, and Myovant.

## Funding Sources

The authors declared that no grants were involved in supporting this work.

## Author Contributions

- Laita Bokhari and Phoebe Cottle − conception of the work, data collection, and final approval of the version to be published.
- Ramon Grimalt, Rodney Sinclair, and Antonella Tosti − conception of the work, critical revision of the article, and final approval of the version to be published.
- Michal Kasprzak − conception of the work, drafting the article, data analysis and interpretation, and final approval of the version to be published.
- Justyna Sicińska − conception of the work, article preparation, and final approval of the version to be published.

## Data Availability Statement

All data are presented within the article.

## Figures and Tables

**Table 1:** Comparison of trichoscopy examination results versus the true difference in hair count

	The measured terminal hair count		The measured change in terminal hair count	True change in terminal hair count (controlled extraction)	Difference between the measured and the true change of terminal hair count	
	manually corrected trichoscopy		H2H matched trichoscopy		
	baseline	follow-up	baseline	follow-up	manually corrected	H2H matched		manually corrected	H2H matched
*1*	*2*	*3*	*4*	*5*	*6*	*7*	*8*	*9*
1	92	91	82	86	−1	4	4	5	0
2	136	128	132	136	−8	4	4	12	0
3	121	118	115	111	−3	−4	−4	1	0
4	152	181	175	179	29	4	3	26	1
5	236	222	258	254	−14	−4	−3	11	1
6	186	208	193	191	22	−2	−2	24	0
7	254	204	261	262	−50	1	−2	48	3
8	205	208	195	199	3	4	3	0	1

Total error in measurement of terminal hair count change in all images								127	6

Average error in measurement of terminal hair count change (relative to total hair count), %								**9±1**	**0.4±0.2**

**Table 2:** Comparison of automatic, manually corrected, and H2H matched PTG results versus the true difference in hair count

	The measured terminal hair count						The measured change in terminal hair count			True change in terminal hair count (controlled extraction)	Difference between the measured and the true change of terminal hair count		
	automatic PTG		manually corrected PTG		H2H matched PTG								
	baseline	follow-up	baseline	follow-up	baseline	follow-up	automatic PTG	manually corrected	H2H matched		automatic PTG	manually corrected	H2H matched
	*1*	*2*	*3*	*4*	*5*	*6*	*7*	*8*	*9*	*10*	*11*	*12*	*13*
1	63	57	143	146	132	133	−6	3	1	2	8	1	1
2	57	75	146	157	144	142	18	11	−2	−3	21	14	1
3	79	72	120	109	108	112	−6	−11	4	4	10	15	0
4	88	89	170	184	174	178	1	14	4	3	2	11	1
5	74	83	145	157	146	152	8	12	6	5	3	7	1
Total error in measurement of terminal hair count change in all images											45	48	4
Average error in measurement of terminal hair count change (relative to total hair count), %											**12±2**	**7±1**	**0.6±0.3**
